# Supplementary material for: Extraction of Tropical Fruit Peels and Development of HPMC Film Containing the Extracts as an Active Antibacterial Packaging Material
Source: Molecules. 2021 Apr 14;26(8):2265. doi: 10.3390/molecules26082265 (PMC8070744; doi:10.3390/molecules26082265)
Supplement: Supplementary file 1 [file molecules-26-02265-s001.pdf]

## Supplementary data

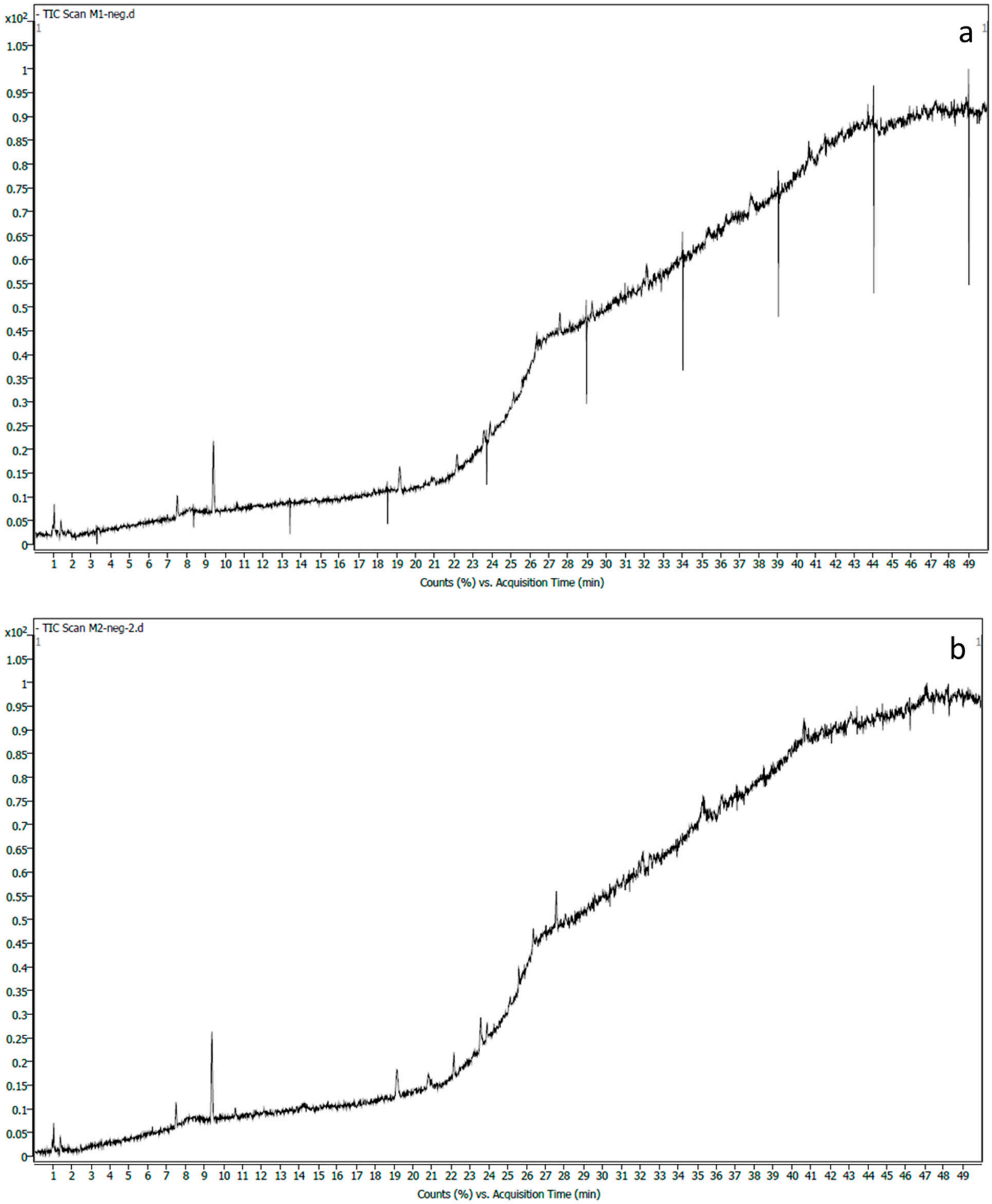


**Figure S1**. Full LC-MS chromatograms of MT-MAE-W/E (a) and MT-Ma-W/E (b) in negative ion mode of analysis.

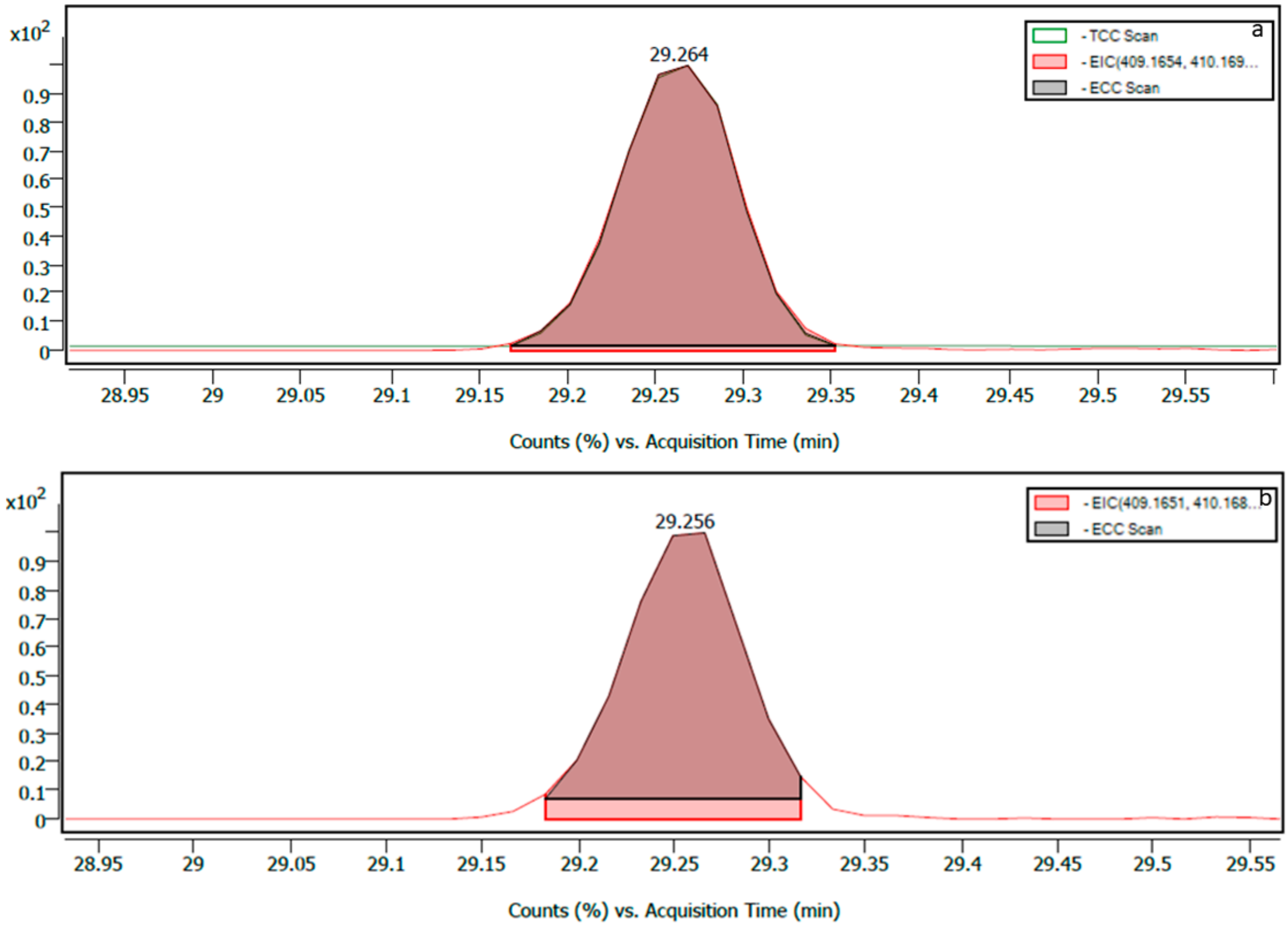


**Figure S2**. LC-MS chromatograms resenting $\alpha$-mangostin of MT-MAE-W/E (a) and MT-Ma-W/E (b).
